# Opening the Door to New Design Rules for Rechargeable Battery Materials

**DOI:** 10.1021/acscentsci.4c00377

**Published:** 2024-03-19

**Authors:** Hala E. Soliman, Amy L. Prieto

**Affiliations:** † Department of Chemistry, Colorado State University, Fort Collins, Colorado 80523-1872, United States; ‡ School of Advanced Materials Discovery, Colorado State University, Fort Collins, Colorado 80523, United States

Rechargeable Li-ion batteries are ubiquitous in most aspects of our daily lives, from small portable devices such as our phones, tablets, and laptops to large applications such as the electrification of transportation. The chemistry of the battery you carry today is essentially unchanged from that of the Li-ion rechargeable batteries commercialized by Sony in the 1990s. While there have been advances in engineering and modifications of the materials used in each aspect of the battery, most battery performance metrics improve only 1 to 2% each year. There is an urgent need for improved energy storage devices to enable growing and emerging markets, but in order to make significant improvements in our batteries, we need new materials and new architectures.

Next-generation batteries will need to store significantly more energy per charge (energy density), be able to charge and discharge very quickly (power density), cycle thousands of times (cycle life), operate over a wide range of temperatures, and be safe, all while being made using inexpensive, scalable manufacturing focused on locally sourced, earth-abundant materials. That is a tall order and one that can not be achieved with current known materials systems! In this issue of *ACS Central Science*, Dincă and co-workers demonstrate a significant breakthrough in two areas: demonstrating that organic cathodes can be effective Li-ion energy storage materials and also eliminating the scarce and expensive cobalt that is in the vast majority of cathode materials used commercially today.^1^ The layered organic cathode they describe could open avenues for new design rules to be considered for electrode materials. Low cost, metal-free tunable materials could also make the battery supply chain more accessible worldwide.

The cathode is just one of three important components of a battery, but it is currently the most significant limitation to the energy density of the overall device.^2,3^ Most current cathode materials are based on transition-metal oxides, where the electrochemical activity of the metal centers enables the storage of lithium ions.^4^ These solid-state materials tend to be electrically conductive and to have low solubility in conventional electrolytes used in batteries, two physical attributes that are important for electrode materials to function long-term in a battery. Organic materials, on the other hand, tend to be insulating and highly soluble in conventional electrolytes. Dincă and co-workers describe electrode materials based on bis-tetraaminobenzoquinone (TAQ), which contains redox-active carbonyl and imine functional groups on a conjugated backbone (Figure 1). This compound can undergo two 2e^–^ redox reactions, which leads to a high theoretical specific capacity for storing Li ions. Through a suite of comprehensive characterization methods, they demonstrate that TAQ is electrically conductive, insoluble in conventional battery electrolytes, has a high specific capacity, and can be used in an active battery with a high weight percentage (which translates to realistic conditions for a full battery).

**Figure 1:**
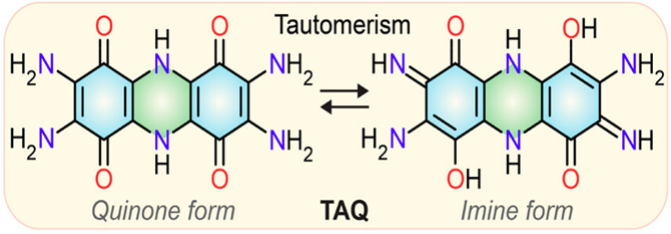
Structure of TAQ, demonstrating the ketol–enol tautomerism. Reproduced with permission from ref (1). Copyright 2024 American Chemical Society.

The Dincă group has been laying the foundation for metal-free organic cathodes for some time. One area of active research that combines the tunability of organic chemistry with the redox capability of metals is based on metal organic frameworks (MOFs). A variety of these compounds have been used as electrode materials in batteries, albeit with limited success.^5^ In an effort to increase the functionality of energy storage materials in a tunable way, the Dincă group has demonstrated that MOFs can be electrically conductive and can be used in a supercapacitor to store charge.^6,7^ However, these compounds still contain metals.

The manuscript described in this *First Reaction* is a significant step beyond MOFs in that there is no metal and the organic moiety can undergo reversible, multielectron redox reactions. This work spans molecular-level characterization (in terms of composition, structure, and transport properties) to electrode and full device characterization. One of the significant challenges in the energy storage community is translating innovation between academic and industrial laboratories.^8^ The Dincă group goes beyond characterizing the structure and transport properties of TAQ electrodes to incorporating the cathodes into commercially relevant slurries that can be assembled into full cells. The TAQ cells are then benchmarked against metrics that enable direct comparison to other cathode material classes (Figure 2). This work enables the development of much more ambitious design rules for high-energy-density cathode materials, which could lead to significant improvement in the overall energy density of future battery chemistries.

**Figure 2:**
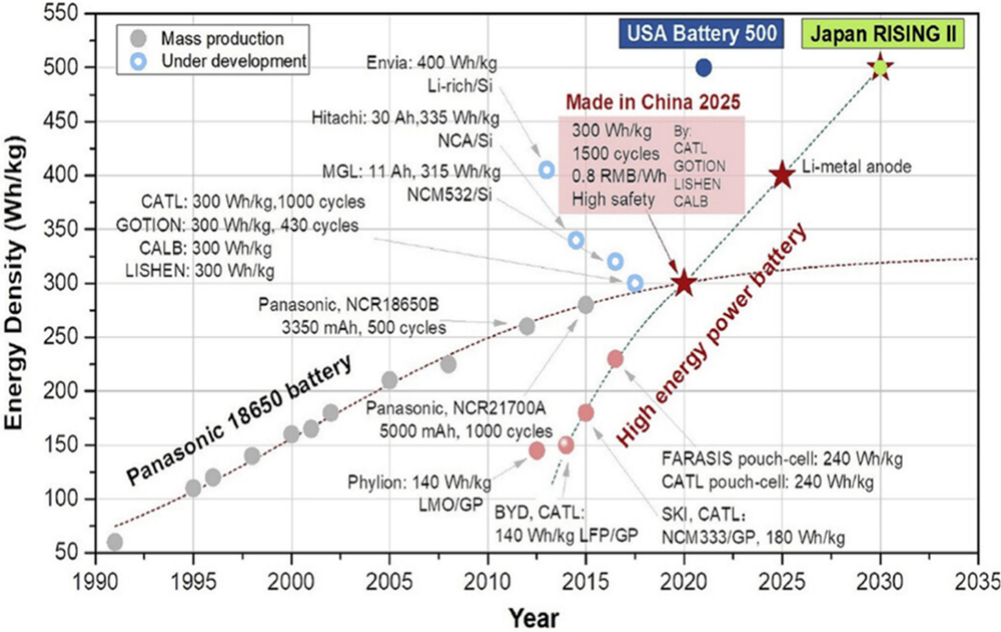
Chronology of energy density improvements in commercialized lithium-ion batteries, with cathodes primarily based on transition-metal oxides. Used with permission from ref (4). Copyright 2023 John Wiley and Sons, Inc.

## References

[ref1] Chen T.; Banda H. ; Wang J. ; Oppenheim J. J. ; Franceschi A. ; Dincâ M. A Layered Organic Cathode for High-Energy, Fast-Charging, and Long-Lasting Li-ion Batteries. ACS Central Science 2024, 10.1021/acscentsci.3c01478. PMC10979494 38559291

[ref2] Kotal M.; Jakhar S.; Roy S.; Sharma H. K. Cathode Materials for Rechargeable Lithium Batteries: Recent Progress and Future Prospects. Journal of Energy Storage 2022, 47, 103534 10.1016/j.est.2021.103534.

[ref3] Chu B.; Guo Y.-J.; Shi J.-L.; Yin Y.-X.; Huang T.; Su H.; Yu A.; Guo Y.-G.; Li Y. Cobalt in High Energy Density Layered Cathode Materials for Lithium ion Batteries. J. Power Sources 2022, 544, 231873 10.1016/j.jpowsour.2022.231873.

[ref4] Xu J.; Cai X.; Cai S.; Shao Y.; Hu C.; Lu S.; Ding S. High-Energy Lithium-Ion Batteries: Recent Progress and a Promising Future in Applications. Energy and Environmental Materials 2023, 6, e12450 10.1002/eem2.12450.

[ref5] Zhu W.; Li A.; Wang Z.; Yang J.; Xu Y. Metal–organic Frameworks and Their Derivatives: Designing Principles and Advances Toward Advanced Cathode Materials for Alkali Metal Ion Batteries. Small 2021, 17 (22), 2006424 10.1002/smll.202006424. 33734586

[ref6] Sheberla D.; Bachman J. C.; Elias J.; Sun C.-J; Shao-Horn Y.; Dincă M. Conductive MOF Electrodes for Stable Supercapacitors With High Areal Capacitance. Nat. Mater. 2017, 16, 220 10.1038/nmat4766. 27723738

[ref7] Day R. W.; Bediako K.; Rezaee M.; Parent L. R.; Skorupskii G.; Arguilla M. Q.; Hendon C. H.; Stassen I.; Gianneschi N. C.; Kim P.; Dincă M. Single Crystals of Electrically Conductive Two-Dimensional Metal–Organic Frameworks: Structural and Electrical Transport Properties. ACS Central Science 2019, 5 (12), 1959 10.1021/acscentsci.9b01006. 31893225 PMC6936098

[ref8] Lin Z.; Liu T.; Ai X.; Liang C. Aligning academia and industry for unified battery performance metrics. Nat. Commun. 2018, 9, 5262 10.1038/s41467-018-07599-8. 30531912 PMC6288112

